# Skin microbiome characterization in acne vulgaris across urban and rural Egyptian populations

**DOI:** 10.3389/fcimb.2026.1816205

**Published:** 2026-06-19

**Authors:** Rana Abdelaal, Nehal Anwar, Ahmed Moustafa, Anwar Abdelnaser

**Affiliations:** 1 Biotechnology Graduate Program, School of Sciences and Engineering, The American University in Cairo, New Cairo, Egypt; 2 Department of Dermatology, Venereology and Andrology, Zagazig University Hospitals, Zagazig University, Zagazig, Egypt; 3 Department of Biology, School of Sciences and Engineering, The American University in Cairo, New Cairo, Egypt; 4 Institute of Global Health and Human Ecology (IGHHE), School of Sciences and Engineering, The American University in Cairo, New Cairo, Egypt

**Keywords:** 16S rRNA, acne vulgaris, *Cutibacterium acnes*, rural, skin microbiome, urban

## Abstract

**Background:**

*Cutibacterium acnes* is recognized as a key contributor to acne, but recent evidence suggests that shifts in skin microbial diversity, rather than simple overgrowth, are critical in disease progression. Despite the unique genetic and environmental characteristics of the Middle East and North Africa, microbiome data on acne remain scarce.

**Objective:**

To characterize the skin microbiome associated with acne vulgaris in Egyptian urban and rural populations and assess the influence of acne severity and lifestyle factors on microbial diversity.

**Methods:**

We recruited 45 acne patients (urban n=37, rural n=8) and 25 healthy urban controls. Skin swabs were collected and analyzed by 16S rRNA sequencing. Microbial community profiles were generated with QIIME2, while differential taxa and functional pathways were evaluated using ANCOM-BC and PICRUSt2.

**Results:**

In urban patients, moderate-to-severe acne was associated with greater microbial evenness and diversity, though species richness was unchanged. Community composition differed significantly by severity. Functional analysis revealed enrichment of amino acid biosynthesis pathways. Rural patients showed greater diversity, distinct microbial structures, and functional enrichment in amino acid and lipid metabolism pathways, with reduced enrichment in energy metabolism pathways. A depletion of *C. acnes* ASVs in patients with more severe acne across both cohorts was observed, possibly indicating a strain-specific behavior of *C. acnes*.

**Conclusion:**

Interventions that selectively eliminate pathogenic *C. acnes* strains while conserving beneficial ones may prove more efficacious for managing acne than broad-spectrum approaches. Environmental context significantly shapes the acne-associated skin microbiome. It influences both the taxonomic composition and potential function.

## 1 Introduction

Acne Vulgaris is a nearly universal disease. According to studies conducted over the past decade, it is the eighth most common disease globally, with prevalence rates of 49.8% to 93.2% (Hay et al., 2014).

The skin is thought to provide nearly 30 m² of diverse microbial habitats for the skin microbiota, a significant factor influencing human skin health. However, bacteria that are typically beneficial to their hosts can become harmful in certain situations. Dysbiosis in the microbiota (Iebba et al., 2016) is associated with skin inflammation and linked to several diseases, including acne. *Cutibacterium acnes* has long been thought to play a central role in the development of acne. *Cutibacterium acnes* has recently been classified into three subspecies: *acnes* (type I), *defendens* (type II), and *elongatum* (type III). Among these, *C. acnes* subsp. *acnes* (type I) are further divided into the subtypes IA1, IA2, IB, and IC (McDowell et al., 2012; Dekio et al., 2019). Our understanding of the role of *Cutibacterium acnes* in the pathophysiology of acne has recently undergone a paradigm shift; a decrease in the diversity between the six phylogenetic groups, rather than sudden *C. acnes* proliferation in the skin appendages, has been associated with acne progression (Dagnelie et al., 2018). Shedding light on the critical role of a tight equilibrium between members of its phylotypes and within the skin microbiota in the development of this skin disease.

Host demographics, genetics, and behavior can alter the skin’s microbial community (Fierer et al., 2008). Despite the unique genetic, environmental, and cultural factors in the Middle East and North Africa (MENA), studies of acne microbiome signatures in this region remain scarce. To address this gap, our study investigates skin microbiome signatures associated with acne vulgaris in two distinct Egyptian populations, urban and rural cohorts, to evaluate how disease severity, geographic and lifestyle differences may influence microbial diversity and acne progression.

## 2 Materials and methods

### 2.1 Recruitment of acne patients and healthy volunteers

The present study recruited 45 patients with acne and 25 healthy volunteers. Acne patients were categorized into urban (n=37) and rural (n=8) groups. Within the urban group, 26 individuals had mild acne, and 11 had moderate-to-severe acne. All rural acne patients were diagnosed with mild-to-moderate acne. Healthy volunteers had no acneiform lesions on the face, chest, or back. A dermatologist determined acne severity according to the 2016 European S3 acne guideline (Nast et al., 2016). Urban skin samples were collected at a single point in time from students at the American University in Cairo over a sample collection campaign in October 2024. The inclusion criteria required that participants had not taken systemic antibiotics for 30 days or used topical antibiotics for 15 days. The exclusion criteria included anyone who had used systemic or topical isotretinoin in the last 30 days, had recent facial peels or laser treatments, had any cosmetic facial procedures, or had active skin conditions such as rosacea, eczema, psoriasis, or other rashes. Before sampling, participants were asked to avoid using any skincare products or facial cleansers the preceding evening. On the sampling day, face washing was not allowed; only water was permitted. Makeup, sunscreen, and other products were not permitted, and participants had to avoid any facial contact with water for at least 3 hours before the procedure. Rural samples were collected from patients recruited at a public hospital in El-Minya governorate in Upper Egypt, in January 2024. The entire process was guided by dermatologists. Two comparative analyses were performed: the first compared acne samples from urban areas with control samples from the same urban setting, while the second compared mild acne samples from urban areas with mild-to-moderate acne samples from rural areas. All subjects voluntarily signed informed consent. All procedures performed in this study involving human participants were in accordance with the Declaration of Helsinki. This study was approved by the Ethical Review Board of the American University in Cairo in accordance with the relevant guidelines and regulations (Case #2023-2024-046).

### 2.2 Sample collection

Samples were obtained by the swab method. The skin surface of each participant’s cheek was swabbed with a sterile swab for 1 minute. Swabs were pre-humidified in a solution of 0.9% NaCl and 0.1% Tween 20. Each swab was immediately placed in a 1.5 mL sterile tube on ice during sample collection and then stored at −80 °C for subsequent DNA extraction. The same researcher collected all samples. No control swabs were used to assess the background noise, which was one of our system’s limitations.

### 2.3 DNA extraction and 16S rRNA gene sequencing

DNA was extracted from skin samples using the QIAamp DNA Mini Kit (Qiagen) following the manufacturer’s instructions. PCR amplification of the V3-V4 hypervariable region of the 16S rRNA gene was conducted. Sequencing was performed using an Illumina NovaSeq platform by Novogene (Hong Kong SAR, China). QIIME 2 version 2024.10 software (Bolyen et al., 2019) was used for subsequent analyses.

### 2.4 Microbiome profiling and analysis

Bacterial community profiles were generated using QIIME2 (v2024.10), and R (v4.4.1) was used to create the visualization. Demultiplexed single-end sequences were quality-filtered and denoised into amplicon sequence variants (ASVs) via DADA2. Representative ASVs were aligned to the Greengenes2 2022.10 reference database using SEPP fragment insertion to construct a rooted phylogenetic tree. Taxonomic classification was performed against the Greengenes2 2024.09 reference. Alpha and beta diversity metrics were calculated at the amplicon sequence variant (ASV) level using rarefied data. Statistical significance of alpha diversity differences was assessed using the Kruskal-Wallis test, while beta diversity comparisons were performed using PERMANOVA (q < 0.05), with Permdisp confirming homogeneity of dispersion. Differential abundance testing for taxa and predicted MetaCyc pathways (PICRUSt2, v2023.9) was conducted using ANCOM-BC (q < 0.05 for urban; q < 0.001 for rural cohorts). Functional pathway identifiers were mapped to descriptive names using the PICRUSt2 metaCyc_pathways_info.txt reference. We implemented a more rigorous false discovery rate (FDR) threshold in the rural cohort to eliminate taxa and predicted functional pathways whose differential abundance could be predominantly driven by environmental variability, thereby increasing confidence that the identified taxa and pathways are more directly linked to acne status rather than to broader environmental factors.

## 3 Results

### 3.1 Study characteristics

Baseline demographic characteristics are presented in **Table 1**. The study included 70 participants distributed across three groups: urban acne (n = 37), urban controls (n = 25), and rural acne (n = 8). The mean age was 21.0 ± 4.11 years in the urban acne group, 22.0 ± 4.13 years in urban controls, and 18.4 ± 2.56 years in the rural acne group. A statistically significant difference in age was observed between the groups (Kruskal-Wallis test, p = 0.02); however, *post-hoc* pairwise comparisons with Benjamini-Hochberg correction indicated that the significant difference was primarily driven by the rural acne and urban control groups. Nevertheless, this comparison was not part of the study’s predefined analytical design. Most participants across all groups were female, accounting for 67.7% (n = 25) in the urban acne group, 84% (n = 21) in the urban controls, and 75% (n = 6) in the rural acne group. Males represented 32.4% (n = 12), 16% (n = 4), and 25% (n = 2) of the urban acne, urban control, and rural acne groups, respectively. No statistically significant differences in gender distribution were identified among the groups (Fisher’s exact test, p = 0.40).

**Table 1:** Demographic and baseline characteristics of study participants.

Variable	Urban acne (n = 37)	Urban control (n = 25)	Rural acne (n = 8)	P-Value
Age (years), mean ± SD	21.0 ± 4.11	22 ± 4.13	18.4 ± 2.56	0.02
Female, n (%)	25 (67.7%)	21 (84%)	6 (75%)	0.40
Male, n (%)	12 (32.4%)	4 (16%)	2 (25%)	0.40

### 3.2 First Cohort: Acne Patients vs. Healthy Controls (Urban Environment)

#### 3.2.1 Alpha Diversity Analysis

**Figure 1:**
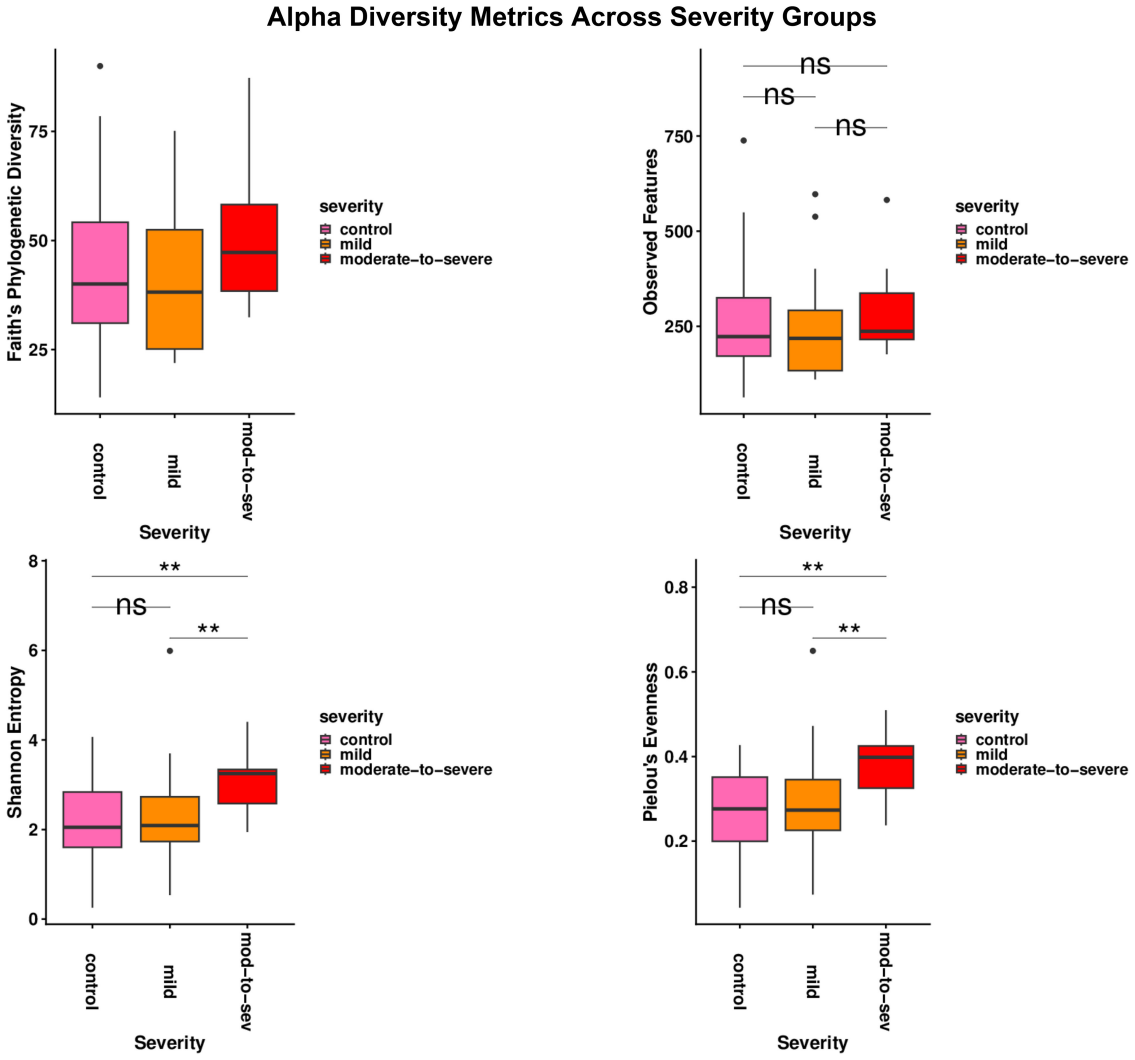
Alpha diversity across acne severity groups and healthy controls. Boxplots show the distribution of four alpha diversity metrics across healthy controls, mild acne, and moderate-to-severe acne groups: **(a)** Faith’s phylogenetic diversity, **(b)** observed features, **(c)** Shannon entropy, **(d)** Pielou’s evenness. Alpha diversity reflects within-sample microbial richness and evenness. ns: not statistically significant (p ≥ 0.05), **: statistically significant difference (p < 0.01).

The Kruskal-Wallis test with Benjamini-Hochberg correction showed significant differences in microbial evenness among severity groups (controls n=25, mild n=26, moderate-to-severe n=11). Pielou’s evenness was highest in the moderate-severe group (0.378 ± 0.076), which was significantly greater than in the controls (0.275 ± 0.102; q=0.0142) and mild cases (0.293 ± 0.111; q=0.0142). Similarly, Shannon entropy was greatest in the moderate-severe group (3.068 ± 0.69), significantly higher than controls (2.20 ± 0.90) and the mild cases (2.30 ± 1.03; both q=0.0085). Nonetheless, no significant differences were observed in Faith_pd or in the observed features among the severity groups (**Figure 1**).

#### 3.2.2 Beta diversity

**Figure 2:**
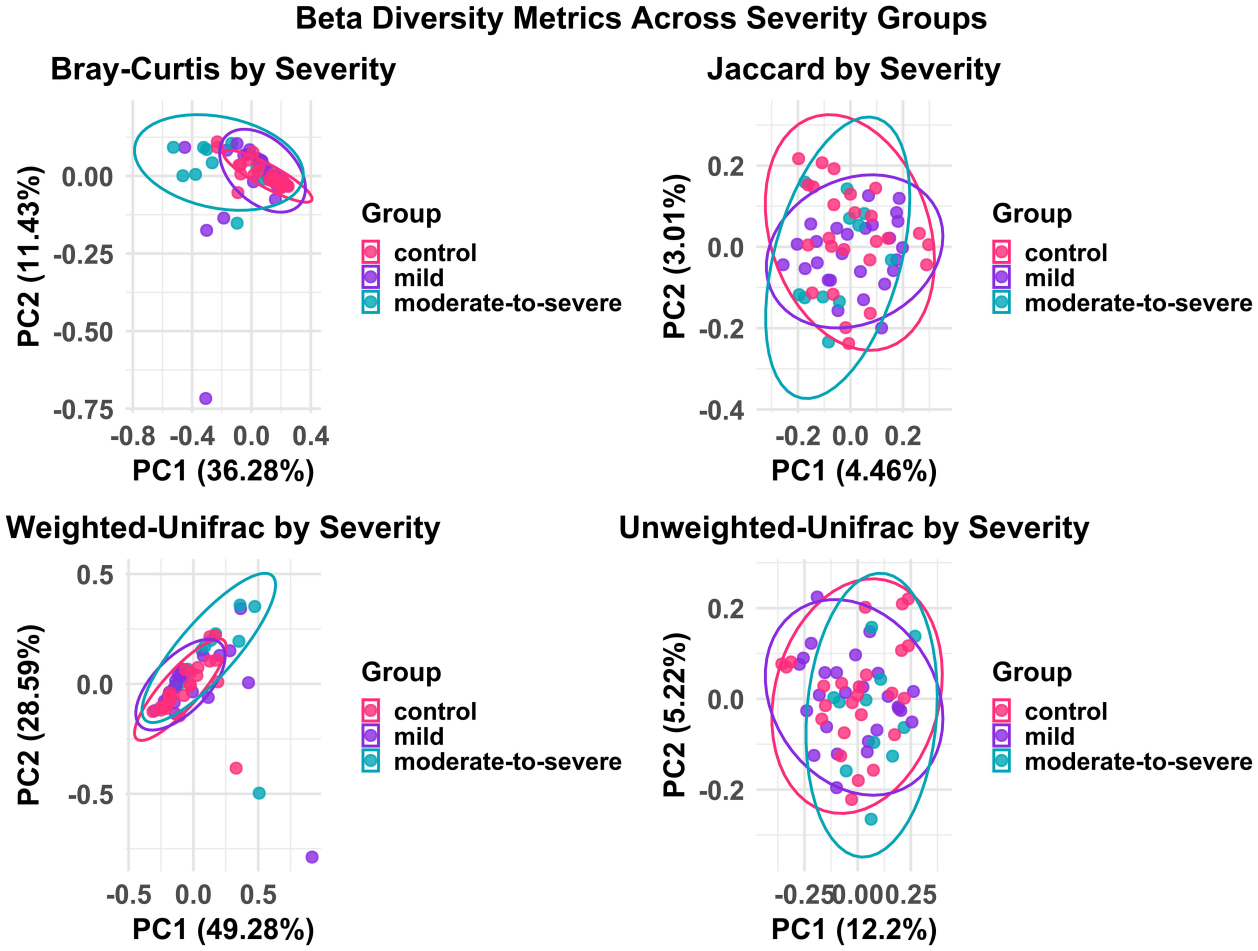
Principal coordinates analysis (PCoA) of beta diversity across severity groups. PCoA plots based on four distance metrics showing beta diversity among healthy controls, mild acne, and moderate-to-severe acne groups. Each point represents a sample, and ellipses indicate within-group dispersion. Statistical significance of group separation was evaluated using PERMANOVA. Panels: **(a)** Bray-Curtis dissimilarity, **(b)** Jaccard distance, **(c)** weighted UniFrac, and **(d)** Unweighted UniFrac.

PERMANOVA analysis showed that the microbial communities were different between the severity groups (controls n=25, mild n=26, moderate-to-severe n=11). The weighted UniFrac distances revealed significant separation between controls and moderate-to-severe cases (R² = 0.165, q = 0.009) and between mild and moderate-to-severe cases (R² = 0.081, q = 0.037). Likewise, based on Bray-Curtis dissimilarity, significant differences were identified between controls and moderate-to-severe cases (R² = 0.17, q = 0.0015), in addition to those between mild and moderate-to-severe cases (R² = 0.10, q = 0.0015). A significant effect of dispersion was noticed for Bray-Curtis distances (PERMDISP, p = 0.048); in contrast, no such effect was observed for weighted UniFrac distances (p = 0.527). No significant differences were observed using the Unweighted UniFrac and Jaccard distance matrices across any of the groups (**Figure 2**).

#### 3.2.3 Differential abundance with ANCOM-BC

**Figure 3:**
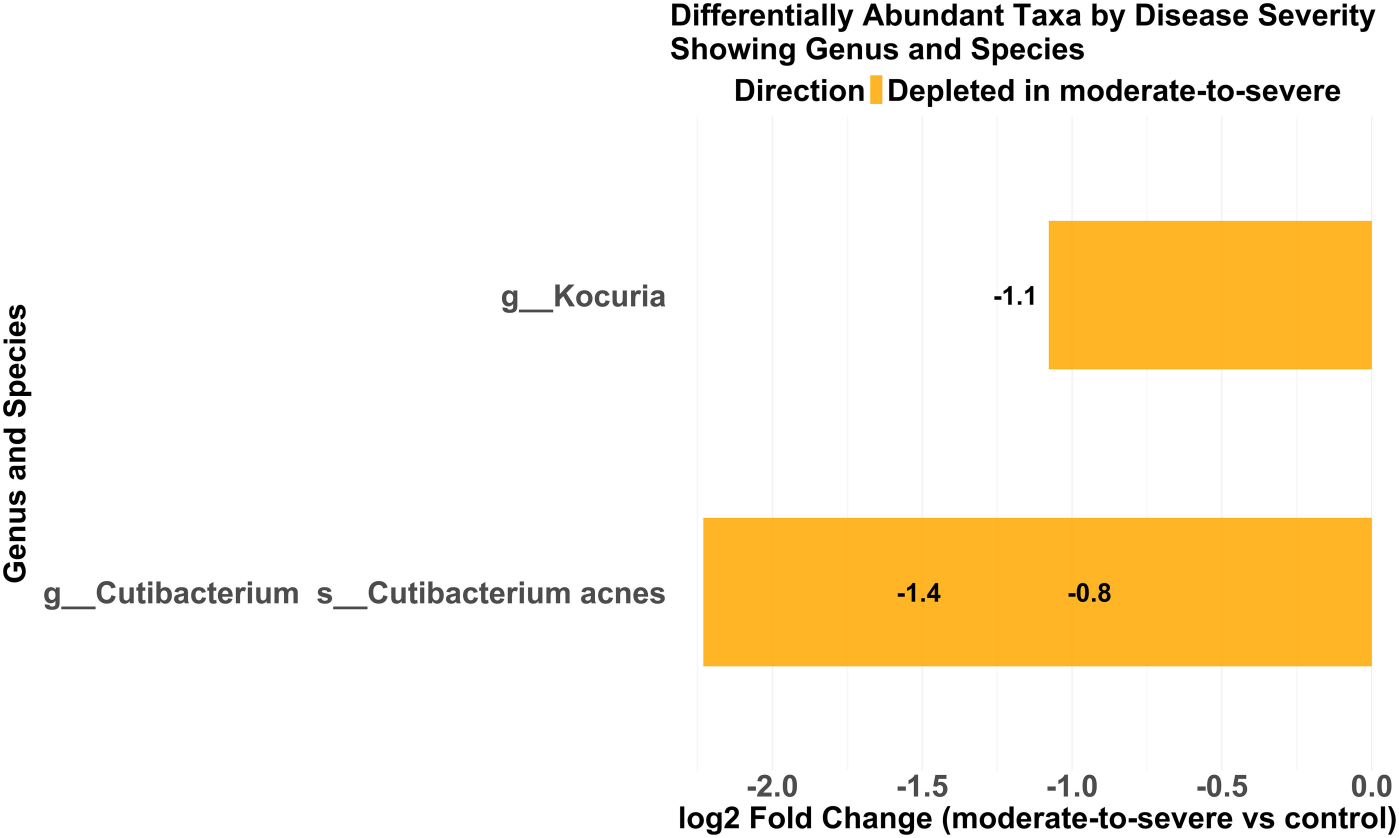
Differentially abundant features identified by ANCOM-BC analysis. Bar plots displaying bacterial taxa with significantly different abundances between groups, as identified by ANCOM-BC analysis. The x-axis shows the effect size, and the y-axis lists the taxa.

ANCOM-BC analysis (controls n=25, mild n=26, moderate-to-severe n=11) revealed three features that were significantly depleted in the moderate-to-severe acne group (n=11). These were *Cutibacterium acnes* ASV1 (logFC = –0.8), *Kocuria* sp. (logFC = –1.1), and *Cutibacterium acnes* ASV2 (logFC = –1.4) (**Figure 3**).

#### 3.2.4 Taxonomic classification

**Figure 4:**
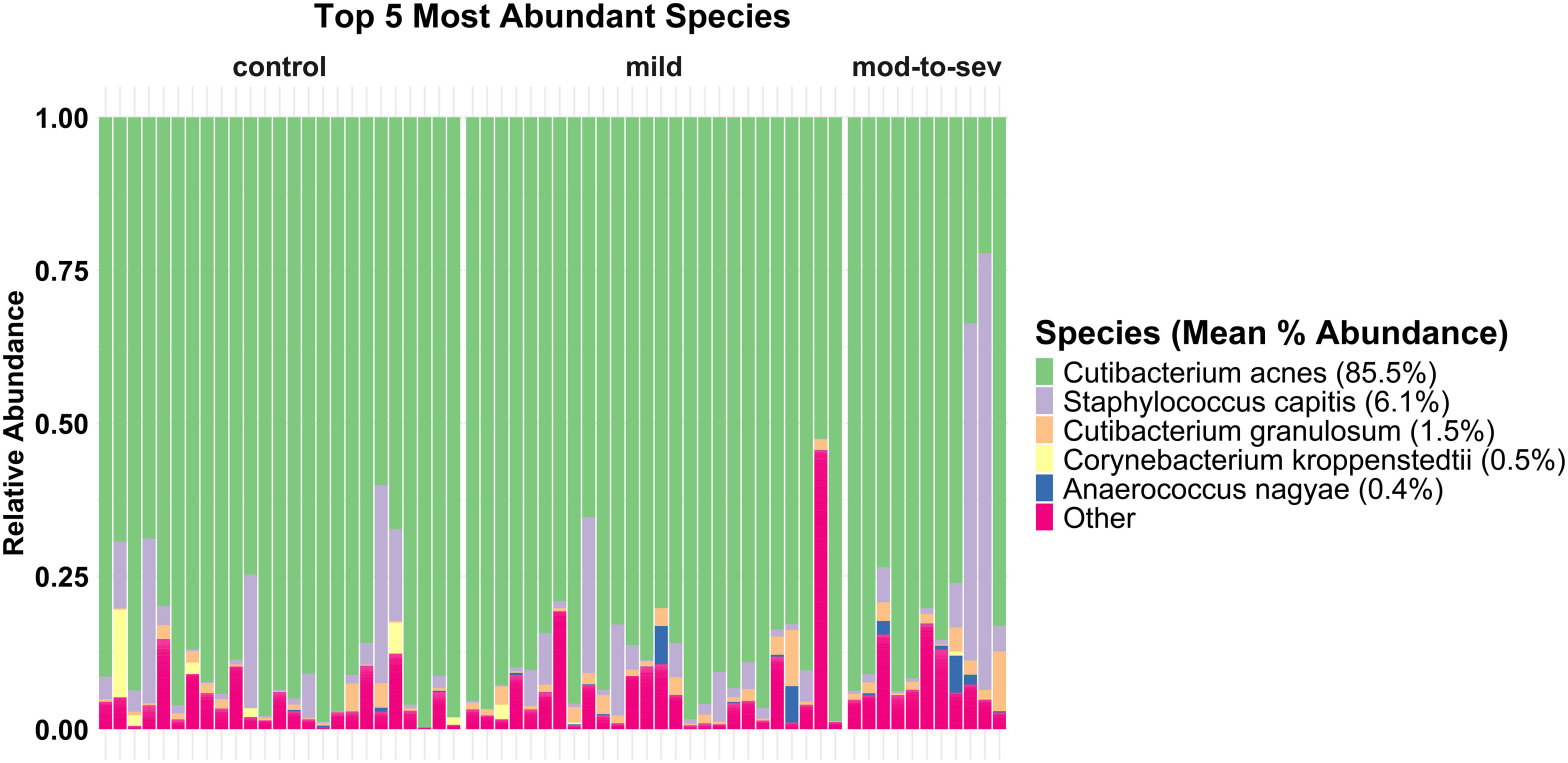
Bacterial community composition across different groups at the species level. Stacked bar plots showing the relative abundance of bacterial taxa at the Species level across healthy controls, mild acne, and moderate-to-severe acne groups. Each bar represents an individual sample, and colors indicate different taxa.

Assignment of bacterial reads at the species level revealed that the five most abundant species across all samples were *Cutibacterium acnes* (85.5%of total reads), *Staphylococcus capitis* (6.1%), *Cutibacterium granulosum* (1.5%), *Corynebacterium kroppenstedtii* (0.5%), and *Anaerococcus nagyae* (0.4%), with *Cutibacterium acnes* being the most dominant. (**Figure 4**).

#### 3.2.5 Mean abundance of *Cutibacterium acnes* across the groups

Species-level analysis indicated no significant differences in the abundance of *Cutibacterium acnes* among the severity groups (controls n=25, mild n=26, moderate-to-severe n=11; Kruskal-Wallis, p = 0.113) (**Supplementary Figure 1**).

#### 3.2.6 Predictive functional profiling using PICRUSt2

**Figure 5:**
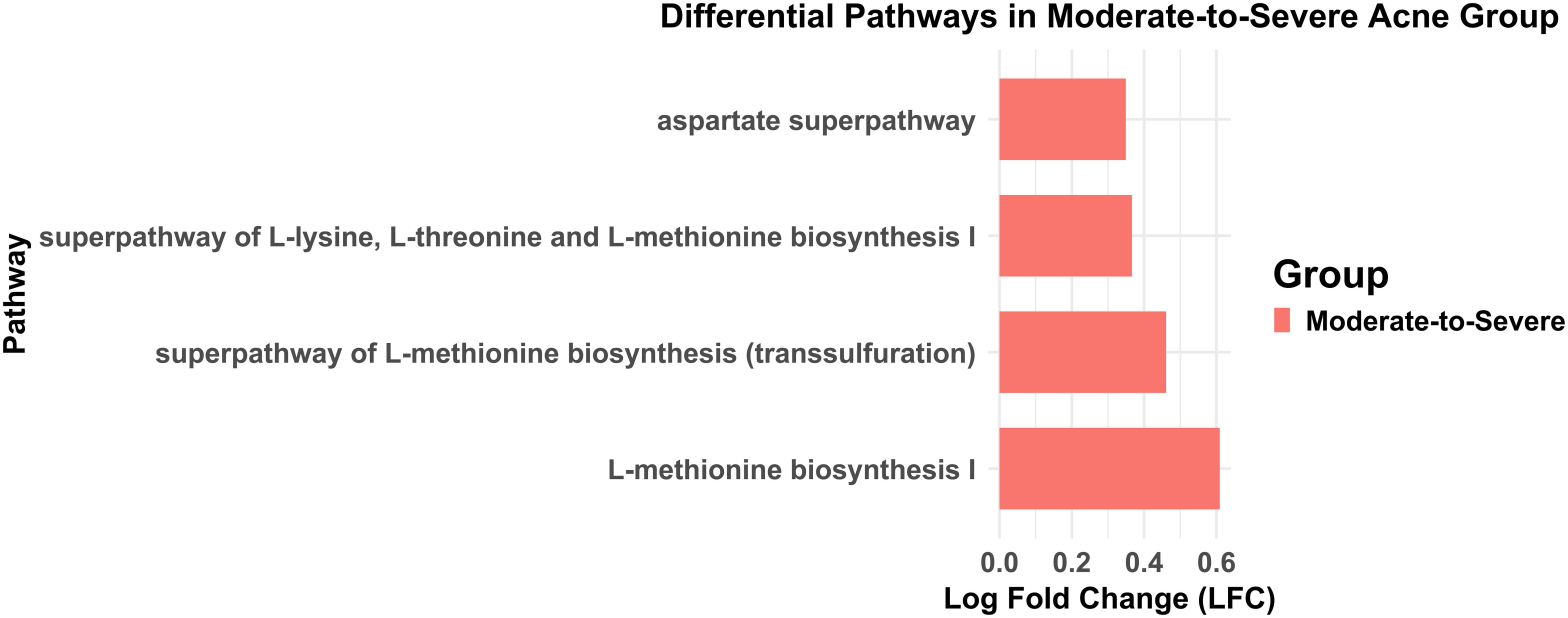
Differentially abundant pathways in the moderate-to-severe acne group. Bar plot showing predicted MetaCyc pathways that were significantly more abundant in the moderate-to-severe acne group, as identified by ANCOM-BC analysis of PICRUSt2 output.

PICRUSt2 analysis (controls n=25, mild n=26, moderate-to-severe n=11) identified four metabolic pathways that were enriched in the moderate-to-severe acne group (n=11). These included the aspartate superpathway, L-lysine/L-threonine/L-methionine biosynthesis I, L-methionine transsulfuration, and L-methionine biosynthesis I (**Figure 5**).

### 3.3 Second cohort: urban acne - rural acne

#### 3.3.1 Alpha diversity

All alpha diversity indices were significantly higher in the rural group (n=8) compared to the urban group (n=26) (q < 0.05), including Faith’s phylogenetic diversity (61.29 ± 21.74 vs 40.24 ± 15.75), observed features (471.37 ± 213.16 vs 241.26 ± 133.68), Shannon diversity (5.88 ± 0.94 vs 2.30 ± 1.035), and Pielou’s evenness (0.671 ± 0.091 vs 0.293 ± 0.111) (**Supplementary Figure 2**).

#### 3.3.2 Beta diversity

**Figure 6:**
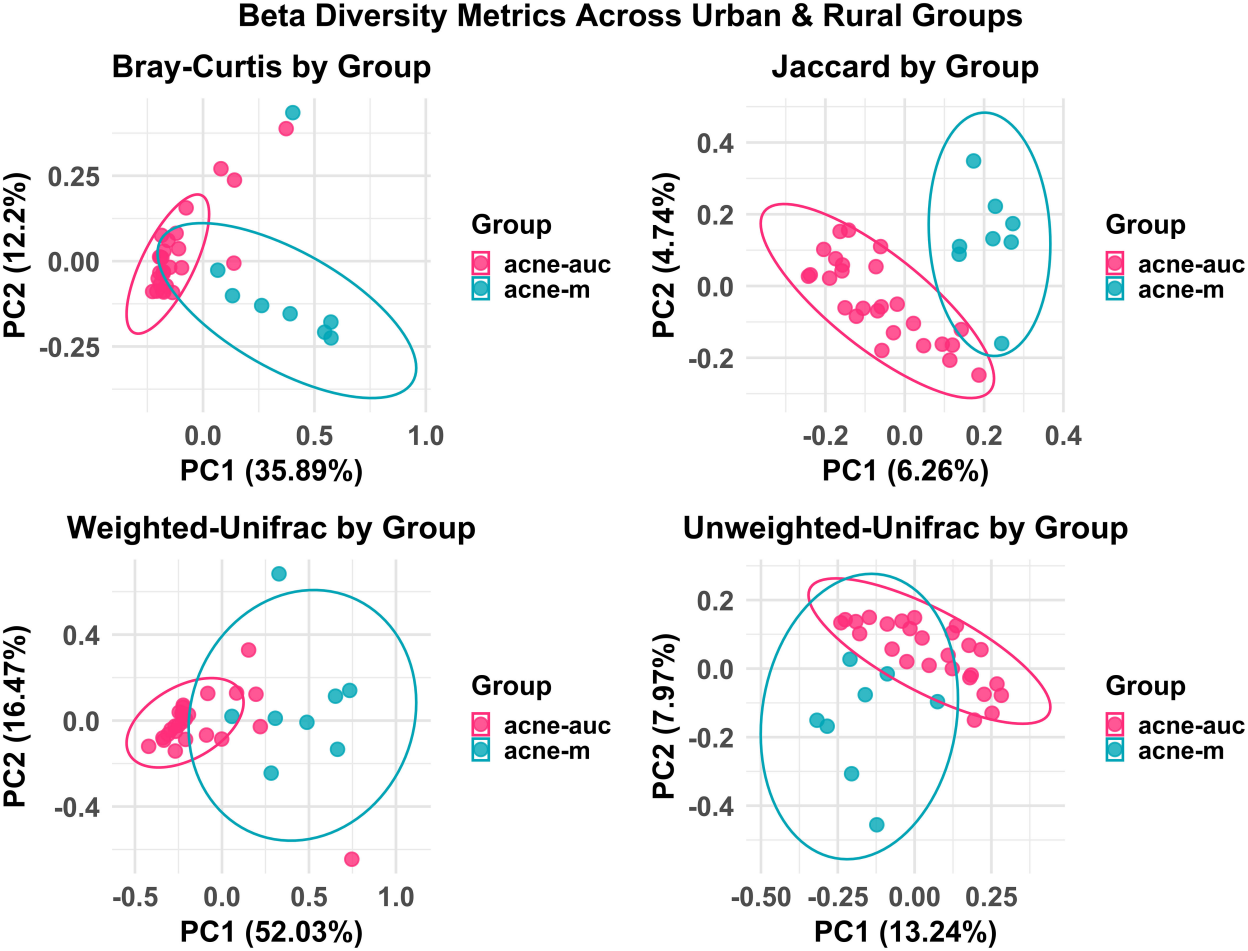
Principal coordinates analysis (PCoA) plots of beta diversity. PCoA based on **(a)** Bray-Curtis dissimilarity, **(b)** Jaccard distance, **(c)** weighted UniFrac, and **(d)** Unweighted UniFrac distances. Each point represents a sample, colored by group, with ellipses indicating the dispersion within each group. These plots collectively illustrate the beta diversity and compositional differences between the urban (acne-auc) and rural (acne-m) groups.

All four distance metrics showed significant differences between rural (n=8) and urban groups (n=26) (PERMANOVA; q=0.001). Dispersion showed a significant difference for Bray-Curtis (PERMDISP p = 0.003, R² = 0.254); however, it showed no significant difference for weighted UniFrac (p = 0.059, R² = 0.290), Jaccard (p = 0.99, R² = 0.052), or unweighted UniFrac (p = 0.05, R² = 0.079) (**Figure 6**).

#### 3.3.3 Differential abundance with ANCOM-BC

**Figure 7:**
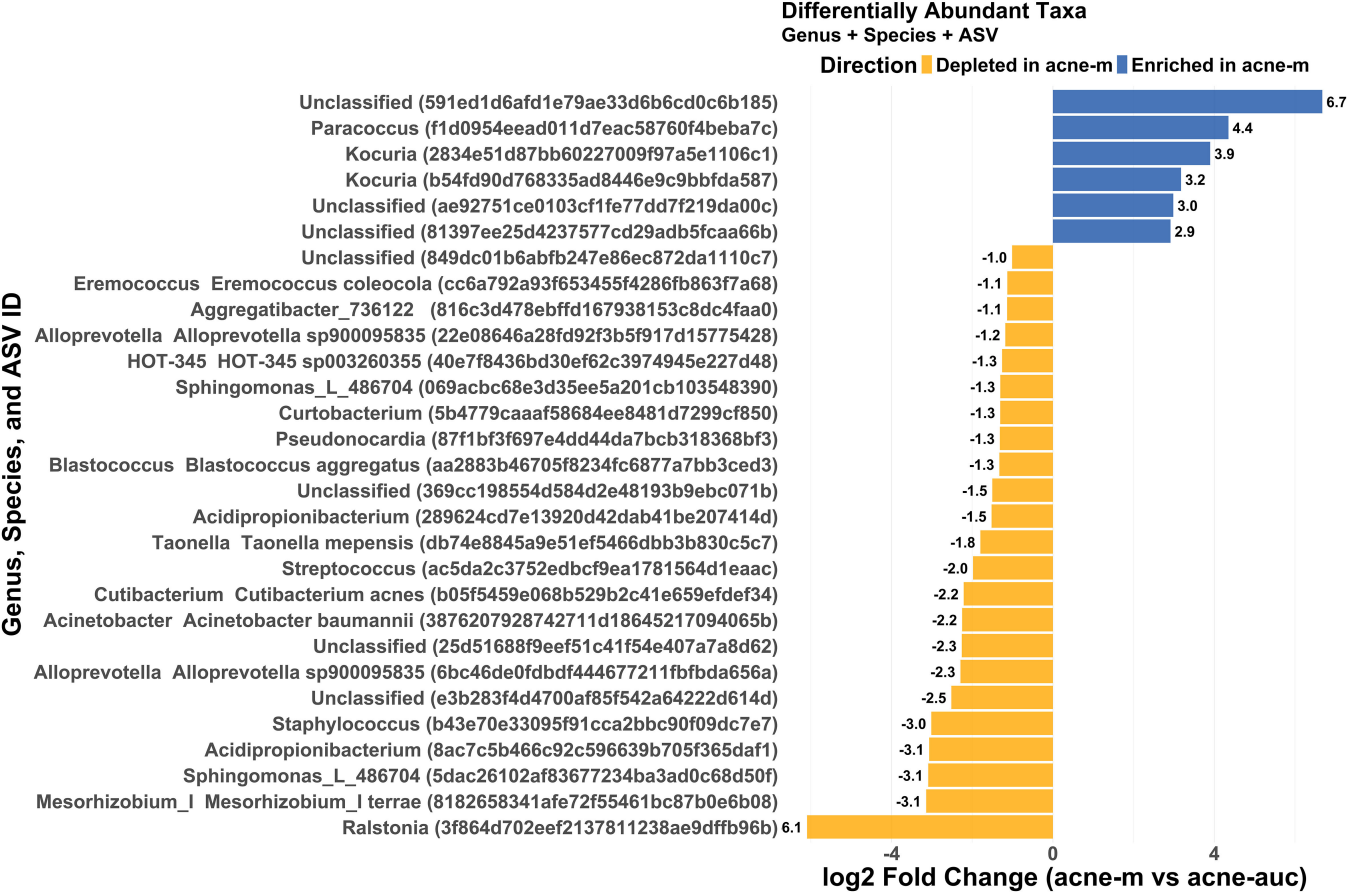
Differentially abundant features identified by ANCOM-BC analysis. Bar plots presenting bacterial taxa with significantly different abundances between the urban (acne-auc) and rural (acne-m) groups, as identified by ANCOM-BC analysis. The x-axis indicates effect size, and the y-axis lists taxa.

ANCOM-BC identified 29 differentially abundant features between rural (n=8) and urban (n=26) acne groups, with 23 features depleted in rural acne (e.g., *Cutibacterium acnes*: logFC = –2.2) and six features enriched in rural acne (e.g., *Kocuria* sp.: logFC = 3.9) (**Figure 7**).

#### 3.3.4 Taxonomic classification

**Figure 8:**
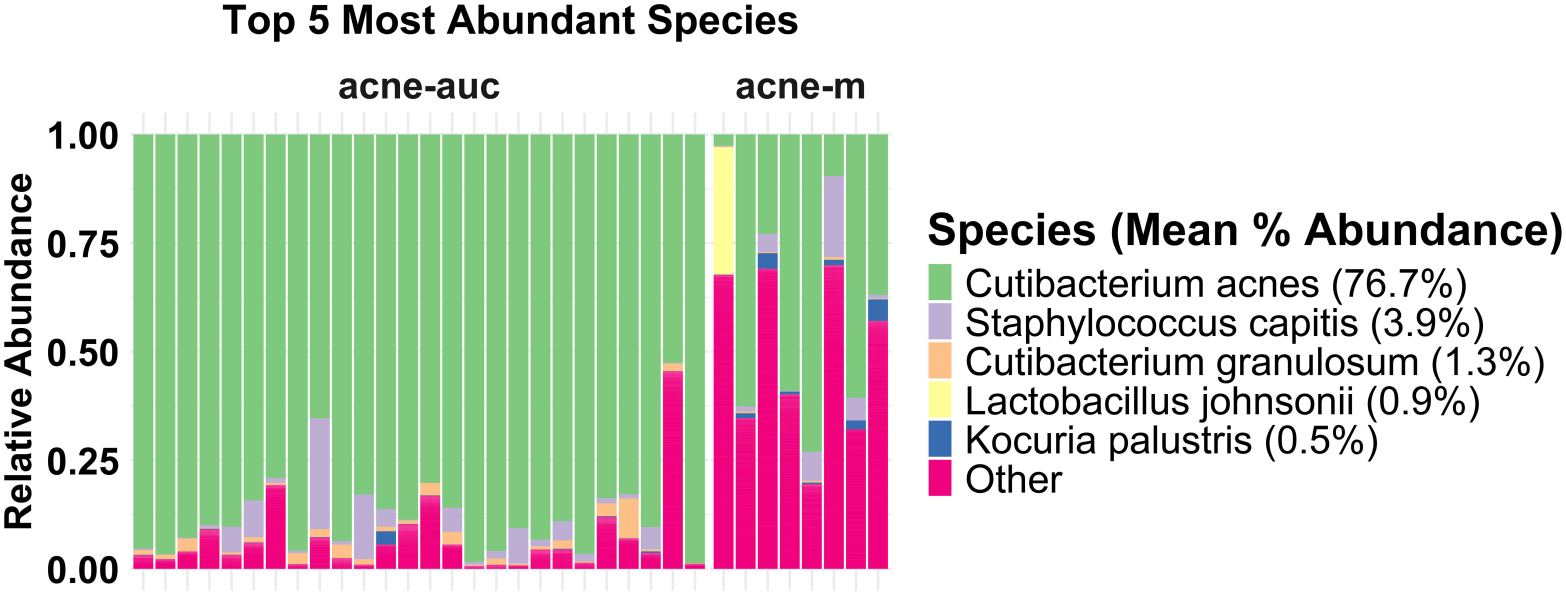
Taxonomic barplots at the species level. Stacked barplots showing the relative abundance of bacterial taxa at the species level across samples from the urban (acne-auc) and rural (acne-m) groups. Each bar represents an individual sample, with colors indicating different taxa.

Taxonomic profiling at the species level revealed that the five most abundant species across all samples were *Cutibacterium acnes* (76.7%)*, Staphylococcus capitis* (3.9%)*, Cutibacterium granulosum* (1.3%)*, Lactobacillus johnsonii* (0.9%), and *Kocuria palustris* (0.5%). (**Figure 8**).

#### 3.3.5 Mean abundance of *Cutibacterium acnes* across the groups

Species-level analysis revealed a significant difference in *Cutibacterium acnes* abundance between rural (n=8) and urban (n=26) groups, with lower abundance in rural samples (41.3%) compared to urban samples (89.6%) (p = 2.093 × 10^-6^) (**Supplementary Figure 3**).

#### 3.3.6 Predictive functional profiling using PICRUSt2

PICRUSt2 analysis (rural n=8, urban n=26) revealed pathway alterations associated with rural acne. A total of 104 pathways were enriched in rural acne, including arginine/polyamine biosynthesis, lipid A biosynthesis, and fatty acid β-oxidation. Inflammatory and immune-related pathways were also enriched in the rural acne group. Conversely, 69 pathways were depleted in rural acne, notably gluconeogenesis, peptidoglycan biosynthesis, and the pentose phosphate pathway (**Supplementary Figure 4**).

## 4 Discussion

Our findings are consistent with prior Chinese research (Li et al., 2019; Shi et al., 2021), demonstrating a correlation between the skin microbiome and acne severity, and indicating a possible microbial-mediated mechanism for acne aggravation and a microbial-based strategy for acne therapy. Significant differences were observed only in the moderate-to-severe acne group, which showed higher alpha diversity when measured with abundance-based metrics, whereas richness-based metrics showed no differences. This indicates that changes in the relative abundance of shared species are the primary drivers of community changes, rather than the presence or absence of unique taxa. Furthermore, rural acne samples exhibited higher alpha diversity and evenness than urban samples, indicating a more balanced microbial community, whereas the urban group was characterized by the predominance of a limited number of taxa.

In the first cohort, differences in beta diversity were identified only using abundance-sensitive metrics in the moderate-to-severe group, suggesting that acne severity modifies relative abundances rather than community membership, with considerable overlap in community composition among groups, as shown in the PCoA plots. The second cohort exhibited significant differences in beta diversity between rural and urban groups across all metrics. The PCoA analysis revealed minimal overlap, demonstrating the considerable influence of lifestyle and environmental factors on microbial composition. Despite some variation in group dispersion, the identification of differentially abundant taxa supports actual biological differences between groups.

*Cutibacterium acnes* is the bacterium most closely linked to acne (Achermann et al., 2014). Here, we observe *C. acnes* ASV depletion in patients with more severe acne across both cohorts. Feature IDs b05f5459e068b529b2c41e659efdef34 and 7d361d9fcb0d7262756088a5f4722a84 were significantly depleted in the moderate-to-severe group compared to controls and mild cases in the urban cohort. The same ASV (b05f5459…) was also depleted in the rural mild-to-moderate group versus the urban mild group in the second cohort. This observation likely demonstrates a strain-specific behavior of *C. acnes*, characterized by a reduction in *C. acnes* strains possibly linked to skin health in acne patients, which we hypothesize facilitates the predominance of more pathogenic *C. acnes* strains (Thompson et al., 2021). However, because *C. acnes* strains cannot be reliably resolved by 16S rRNA sequencing, these strain-specific interpretations remain speculative. The fact that the species’ relative abundance remained constant across severity groups in the first cohort supports our hypothesis, suggesting strain-level shifts drive acne severity. In contrast, the statistically significant reduction in the relative abundance of *C. acnes* in the rural acne group compared with the urban acne group in the second cohort is unlikely to be directly related to acne pathogenesis. But rather attributed to increased microbial diversity in rural environments (Ogai et al., 2022; Durack et al., 2024), which likely introduces interspecies competition among bacteria (Kong et al., 2012), in which competitive species outcompete *C. acnes* on the skin, thereby decreasing its relative abundance (Fitz-Gibbon et al., 2013; Cavallo et al., 2022). Among all the differentially abundant taxa associated with environmental variation, the same *C. acnes* ASV was consistently depleted in both cohorts, reinforcing its potential relevance to acne pathogenesis in the Egyptian population. However, additional environmental parameters, outdoor exposure data, and skin care habits were not available for the current cohorts. In addition, differences in temperature, humidity, and ultraviolet radiation intensity between the October 2024 and January 2024 sampling periods may have influenced skin microbial composition independently of urban-rural differences. Therefore, seasonal, geographic, and environmental factors represent potential confounders and should be addressed in future longitudinal studies with more comprehensive metadata collection.

In our first cohort, the urban moderate-to-severe acne group showed *Kocuria* depletion, whereas the rural mild-to-moderate group in the second cohort showed enrichment. While *Kocuria* species are common skin commensals (Grice et al., 2008; Ogai et al., 2022), a study of acne patients found that *Kocuria varians* was present in a subset of cases, suggesting it can be isolated from acne lesions. However, it was much less common than other bacteria, such as *Staphylococcus* species (Al-Hasseny, 2022); its presence in rural acne samples may reflect environmental exposure, such as contact with soil, plants, or other natural microbial sources (Hospodsky et al., 2014; Ying et al., 2015; Ogai et al., 2022; Zhu et al., 2022). The enrichment of the *Kocuria* genus in our rural cohort reflects findings from the Yanomami rural population, where transitioning to an industrialized lifestyle led to reduced skin microbiome diversity and the loss of several key bacterial genera, including *Kocuria* (Durack et al., 2024).

The *Peptostreptococcaceae* family was enriched in rural acne patients of the second cohort. At the same time, *Peptostreptococcus* species have been linked to elevated levels of *C. acnes* type IA strains, suggesting an association with inflammation (Kwon et al., 2013).

The second dataset, representing the rural acne group with greater acne severity, exhibited a depletion of 23 taxa, including *Ralstonia*. The abundance of this genus changes with acne severity. Specifically, studies show that *Ralstonia* is present at higher levels in healthy and mildly acneic skin, but its abundance decreases in severe acne. This pattern suggests that *Ralstonia* might play a role in maintaining skin homeostasis, and its reduction could be associated with a disrupted skin microbiome in more severe acne (Zhu et al., 2022; Guo et al., 2023), supporting its depletion in more severe rural acne in our data.

The genus *Staphylococcus*, which includes species such as *S. aureus* and the commensal *S. epidermidis*, was depleted in the rural cohort. Although the role of S. e*pidermidis* in acne remains unclear, numerous studies indicate that it may be crucial for regulating *C. acnes* proliferation, particularly the virulent type IA1, and for restoring skin dysbiosis (Wang et al., 2014; Brandwein et al., 2016; Xia et al., 2016; Dreno et al., 2017).

None of the remaining depleted taxa in rural acne patients, compared with their urban counterparts, has been directly associated with the pathogenesis or severity of acne in the existing literature. The depletion may arise from lifestyle variations, environmental microbial exposure, and interspecies competition among bacteria (Kong et al., 2012) (Dagnelie et al., 2019). Rural settings expose individuals to a variety of microbes (eg, soil, plants, animals, livestock, and natural water sources), promoting a more complex skin microbiome (Durack et al., 2024). In contrast, urban settings reduce microbial diversity and favor dominant acne-associated taxa such as *Cutibacterium acnes* (Myers et al., 2024). The greater microbial diversity observed in our rural acne cohort likely supports the presence of competitive environmental taxa that may surpass the identified depleted taxa on the skin through interspecies competition (Dagnelie et al., 2019). The urban skin microbiome, on the other hand, may be a better place for these taxa to live and grow because of its lower biodiversity.

The moderate-to-severe acne group in the urban cohort exhibited an enrichment in four amino acid biosynthesis pathways, including the aspartate superpathway, L-lysine/L-threonine/L-methionine biosynthesis I, methionine transsulfuration, and methionine biosynthesis I. On the other hand, enriched pathways in the rural cohort were mainly associated with amino acid metabolism and lipid and fatty acid metabolism. Inflammatory and immune-related pathways were also enriched in the rural acne group. On the other hand, depleted pathways included those associated with energy metabolism and peptidoglycan and cell wall biosynthesis. Alterations in microbial amino acid metabolism have been observed in patients with severe acne within a Chinese cohort; these findings suggest potential links to acne pathogenesis (Guo et al., 2023). Consistent with our findings, the Yanomami rural microbiomes exhibited distinct functional pathways associated with lipid metabolism, which emphasizes the role of the environment in microbiome modulation (Ying et al., 2015; Ogai et al., 2022; Durack et al., 2024). Since PICRUSt2 was used to create these functional inferences, they represent predicted functions rather than direct measurements from metagenomic or metabolomic analyses. Accordingly, these interpretations should be treated with caution.

## 5 Conclusion

Our results indicate that the skin microbiome exhibits significant plasticity, with environmental context influencing both taxonomic structure and functional pathways. A detailed understanding of *C. acnes* ecology suggests that treatments that selectively eliminate pathogenic strains while preserving beneficial ones could be more effective than broad-spectrum methods. This may involve the application of strain-targeted phage therapy, topical prebiotics that enhance commensal phylotypes, or live probiotic skincare containing beneficial isolates. These precision strategies may mitigate the risk of antibiotic resistance (Cai et al., 2026) and minimize disruption of the skin microbiome, potentially resulting in improved long-term outcomes for acne patients. Moreover, our data from the rural population demonstrate that extended exposure to environmental microbes can modify the adult human skin microbiome and integrate these taxa into the existing community. This emphasizes the potential of continuous topical microbial supplementation to reshape the skin microbiome and significantly improve skin health (Durack et al., 2024), while also highlighting the need to account for environmental and lifestyle factors when tailoring acne management strategies for diverse patient groups. A limitation of this study is the small sample size of the rural cohort (n = 8) compared with the urban (n = 37) and control (n = 25) groups, which reduces statistical power and limits the robustness of inter-group comparisons. In addition, the lack of stratification by acne severity in the rural cohort limits matched comparisons between populations. Although conservative statistical criteria (q < 0.001) were applied, the findings should still be interpreted cautiously, particularly regarding generalizability beyond the studied cohorts. The absence of negative control swabs in the present study is another limitation. However, all sample collection and processing operations were carried out using sterile, standardized methods, including sterile swabs, gloves, and consistent extraction techniques, to reduce the risk of contamination. The lack of a rural healthy control group limits how our results can be interpreted, as it remains challenging to distinguish microbial changes linked to acne from broader environmental, geographic, seasonal, or climatic variations in the skin microbiome. We therefore highlight the need for future studies, including appropriate rural healthy controls, to strengthen causal inference.

## Data Availability

The dataset generated in this study has been deposited in the NCBI BioProject database under accession number PRJNA1400918.

## Supplementary material

Supplementary Methods Describe the detailed procedures for DNA extraction, 16S rRNA gene sequencing, microbiome data processing, and downstream analyses performed using QIIME 2 and R. This includes data import, sequence quality assessment, denoising and ASV generation using DADA2, phylogenetic tree construction via SEPP, taxonomic classification using a Greengenes-based naïve Bayes classifier, alpha and beta diversity analyses, differential abundance testing using ANCOM-BC, and predictive functional profiling using PICRUSt2.
