# Supplementary material for: Skin microbiome characterization in acne vulgaris across urban and rural Egyptian populations
Source: Front Cell Infect Microbiol. 2026 Jun 19;16:1816205. doi: 10.3389/fcimb.2026.1816205 (PMC13328423; doi:10.3389/fcimb.2026.1816205)
Supplement: Supplementary Methods — Describe the detailed procedures for DNA extraction, 16S rRNA gene sequencing, microbiome data processing, and downstream analyses performed using QIIME 2 and R. This includes data import, sequence quality assessment, denoising and ASV generation using DADA2, phylogenetic tree construction via SEPP, taxonomic classification using a Greengenes-based naïve Bayes classifier, alpha and beta diversity analyses, differential abundance testing using ANCOM-BC, and predictive functional profiling using PICRUSt2. [file SupplementaryFile1.pdf]

Skin Microbiome Characterization in Acne Vulgaris across Urban and Rural Egyptian Populations

|

Rana Abdelaal[1], Nehal Anwar[2], Ahmed Moustafa[3], and Anwar Abdelnaser[4*]

[1] Biotechnology Graduate Program, School of Sciences and Engineering, The American University in Cairo, New Cairo, 11835, Egypt.

[2] Department of Dermatology, Venereology and Andrology, Zagazig University Hospitals, Zagazig University, Egypt

[3] Department of Biology, School of Sciences and Engineering, The American University in Cairo, New Cairo, 11835, Egypt.

[4] Institute of Global Health and Human Ecology (IGHHE), School of Sciences and Engineering, The American University in Cairo, New Cairo, 11835, Egypt.

* Corresponding Author:

Anwar Abdelnaser – Institute of Global Health and Human Ecology (IGHHE), School of Sciences and Engineering, The American University in Cairo, New Cairo, 11835, Egypt; Email: anwar.abdelnaser@aucegypt.edu

Supplementary figures:

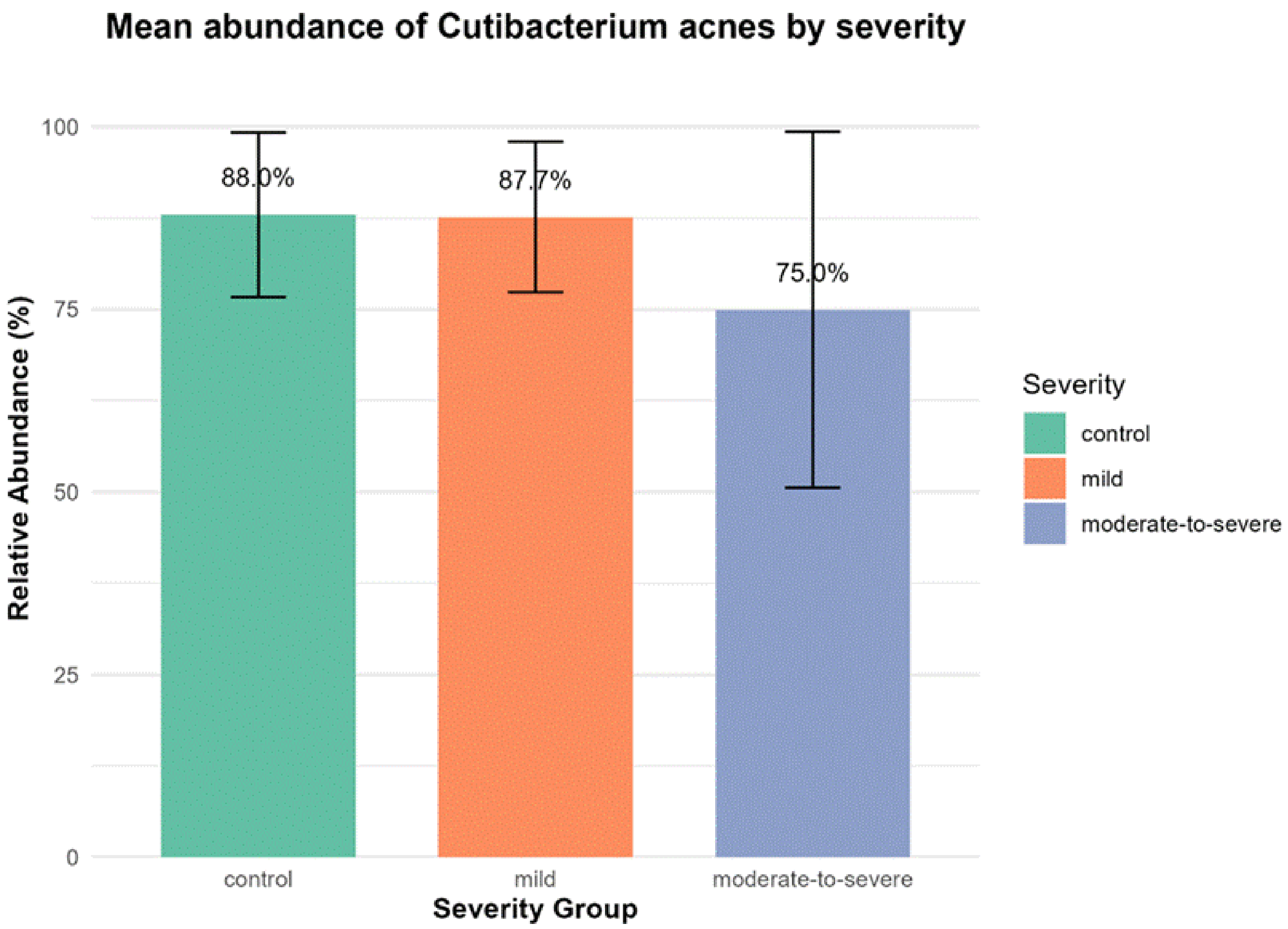

Supplementary Figure 1 | Mean Abundance of *Cutibacterium acnes* Across the Three Severity Groups | Bar plot showing the mean relative abundance of *Cutibacterium acnes* in healthy controls, mild acne, and moderate-to-severe acne groups. The plot highlights differences in the prevalence of this species across acne severity levels.

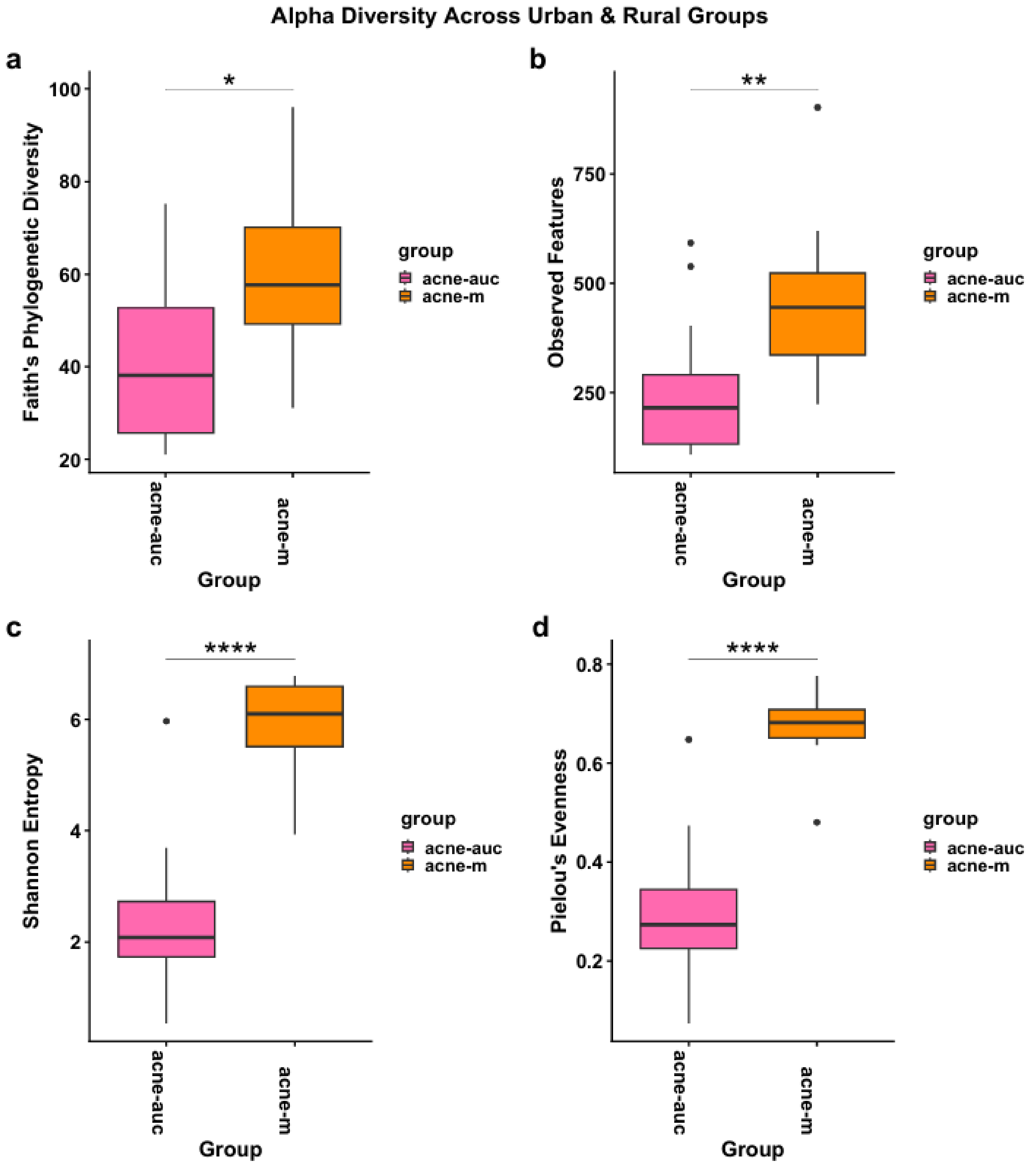

Alpha Diversity Across Urban & Rural Groups
a
b
c
d
*
**
****
****
Faith's Phylogenetic Diversity
Observed Features
Shannon Entropy
Pielou's Evenness
100
80
60
40
20
750
500
250
6
4
2
0.8
0.6
0.4
0.2
acne-auc
acne-m
Group
group
acne-auc
acne-m

Supplementary Figure 2 | Alpha Diversity Across Groups | a) Faith’s phylogenetic diversity, b) Observed features, c) Shannon entropy, and d) Pielou’s evenness are shown for urban (acne-auc) and rural (acne-m) groups. Boxplots indicate the distributions of phylogenetic diversity values (Faith’s PD), counts of observed features (richness), Shannon entropy, and Pielou’s evenness across the groups, demonstrating within-sample microbial diversity and evenness.

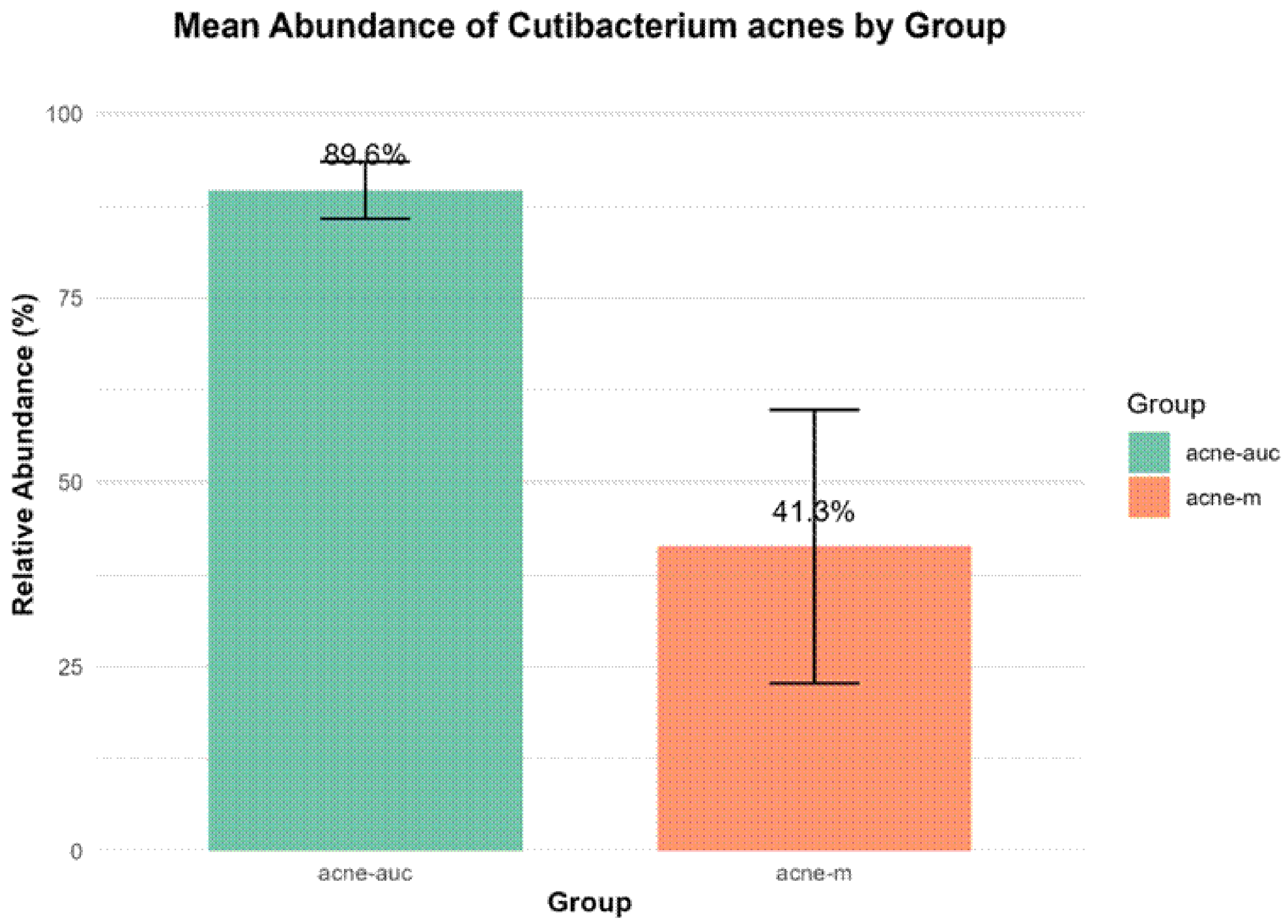

Supplementary Figure 3 | Mean Abundance of *Cutibacterium acnes* Across the Urban and Rural Groups | Bar plot showing the mean relative abundance of *Cutibacterium acnes* in urban (acne-auc) and rural (acne-m) groups. The plot highlights differences in the prevalence of this species across the two groups.

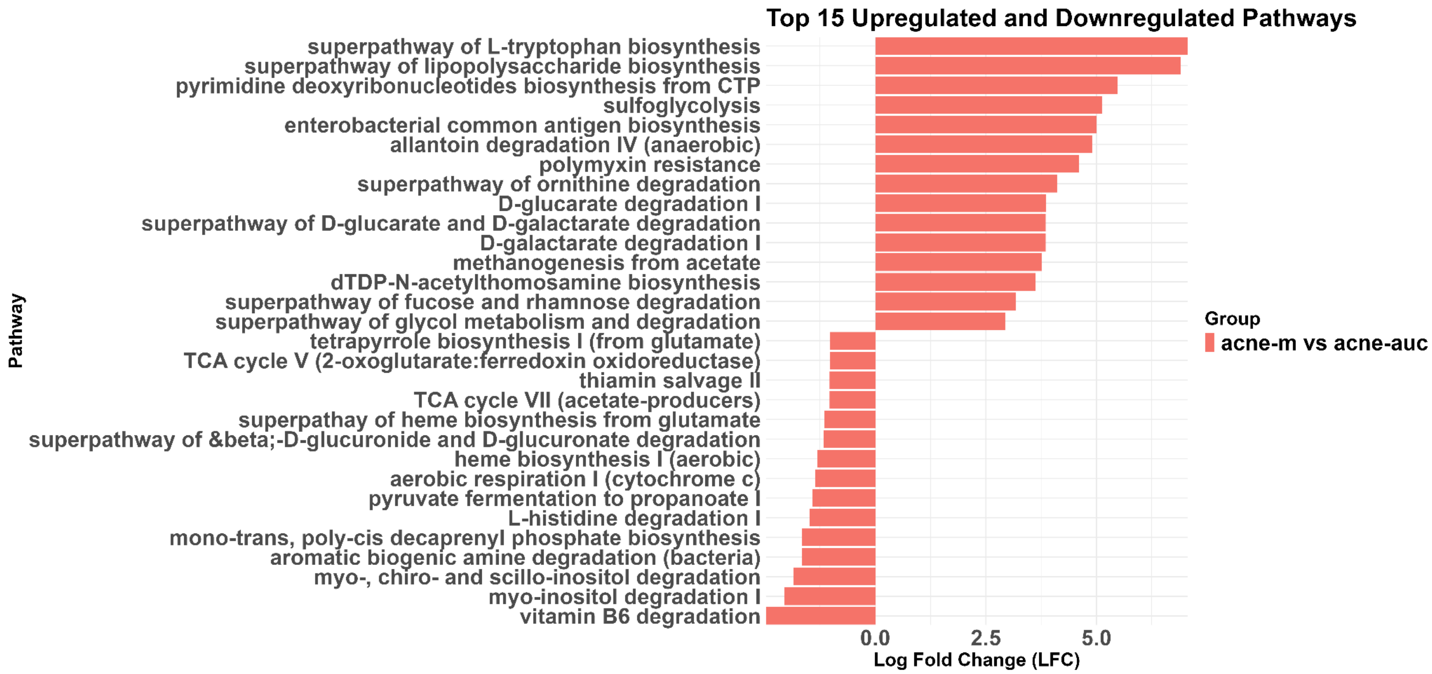

Supplementary Figure 4 | Differentially Abundant Pathways Identified by ANCOM-BC after PICRUST2 Analysis | Bar plots displaying the top 15 upregulated and downregulated predicted MetaCyc pathways that are significantly different between urban (acne-auc) and rural (acne-m) groups, as identified by ANCOM-BC after PICRUST2 analysis. The x-axis shows the effect size, and the y-axis lists the pathway.

Supplementary Materials and Methods:

- **Microbiome Data Processing and Analysis**

QIIME 2 version 2024.10 was used to generate the QZA artifacts for microbiome data analysis. Subsequently, R version 4.4.1 was employed for exporting the QZA files and generating the visualizations to interpret the results.

- **Importing data into Qiime2**

Single-end, demultiplexed sequencing data in Fastq format were imported into Qiime2 using a manifest file. The quality of the DNA sequencing run was assessed through the number of sequences obtained per sample and the distribution of the sequence quality score at each position.

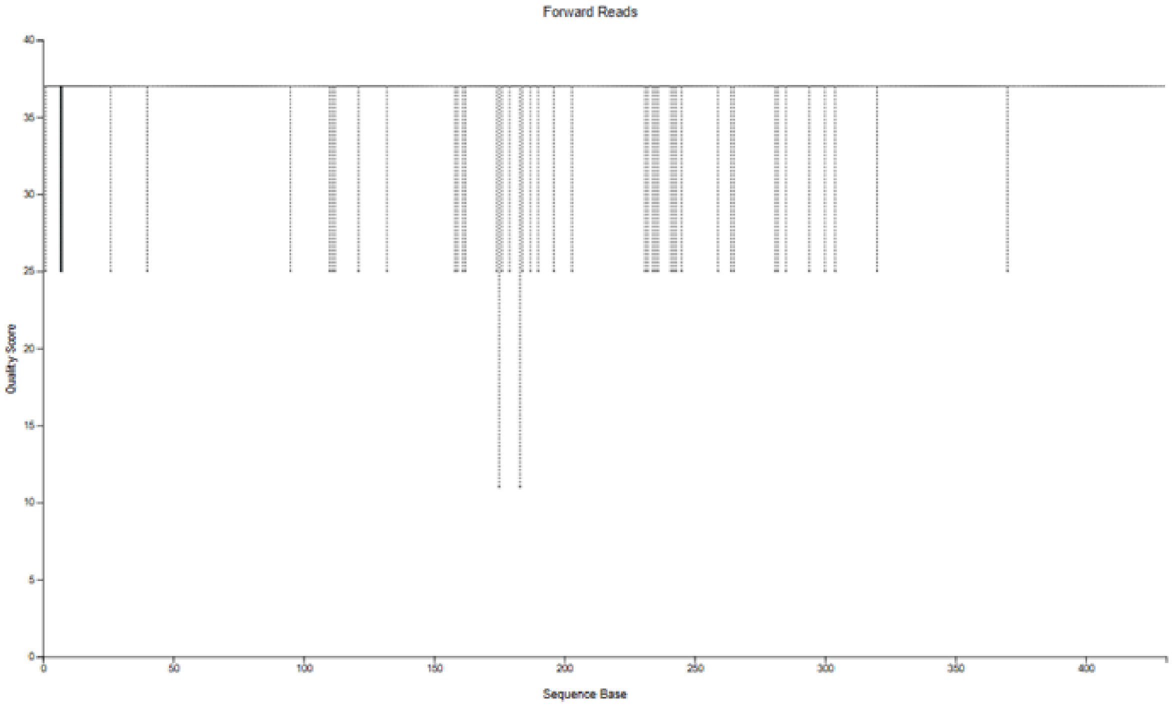

a)

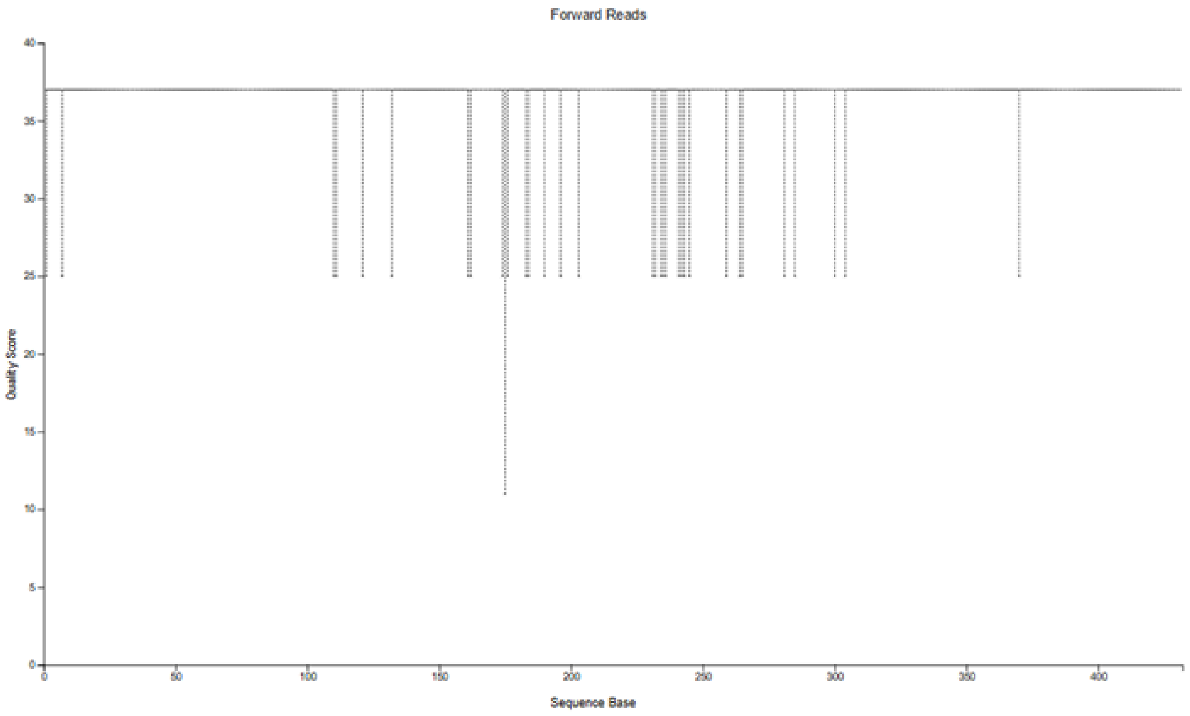

b)

Qiime2 visualization of the sequence quality plot | shows a boxplot of the quality score distribution for each position of the input sequences. a) The first comparison of acne samples from urban areas with control samples from the same urban setting. b) The second comparison of acne samples from urban and rural geographical locations.

- **Sequence quality control and feature table construction**

For sequence quality control and feature table construction, denoising was done via the DADA2 plugin (Callahan et al., 2016), where features (amplicon sequence variants -ASVs-) were generated. The quality plot shows a high quality score along the full length of the reads. Therefore, the reads were truncated at a 400 bp position.

- **Generating a phylogenetic tree**

The fragment insertion tree building method (Janssen et al., 2018) using the SEPP action was used. This method aligned the representative sequences that were generated during the denoising step to full-length known sequences in the Greengenes 2022.10 backbone SEPP reference tree, to create a fixed rooted phylogenetic tree.

- **Alpha rarefaction plots**

Data normalization was performed to account for the uneven sampling depth between the samples. The rarefaction normalization method was used, where a sampling depth was selected to minimize the samples lost while maximizing the sequences retained for diversity analysis.

- **Taxonomic classification**

A pre-trained naive Bayes machine-learning classifier that was trained to differentiate taxa present in the Greengenes2 2024.09 full-length sequences reference set was used to predict the taxonomic affiliation of each ASV. Then, a taxonomic bar chart was built to visualize the taxonomic composition of the samples.

- **Diversity analysis**

The pipeline action core-metrics-phylogenetic was utilized within the Qiime2 software to compute several commonly used alpha and beta diversity metrics and generate principal coordinate analysis (PCoA) plots using the EMPeror visualization tool for each of the beta diversity metrics. (Vázquez-Baeza et al., 2013) Statistical tests were conducted to assess whether within-sample (alpha) diversity significantly differed across the groups, based on boxplots and non-parametric Kruskal-Wallis test results. Microbiome community structure was compared between groups using beta diversity, with visual inspection of principal coordinates analysis (PCoA) plots. Statistical significance of differences between groups was evaluated using the PERMANOVA test, with significance determined based on q-values (adjusted p-values) to account for multiple testing. To validate that the groups differ in centroids (multivariate means), not in dispersions (variances), significant differences in PERMANOVA (P-value < 0.05) were confirmed using the Permdisp statistical test (P-value > 0.05), to rule out that the significant differences found by PERMANOVA are due to the high degree of variation (dispersion) within one of the groups of interest.

- **Differential abundance testing**

The ANCOM-BC package was used to identify individual taxa whose relative abundances are significantly different across groups (Lin & Peddada, 2020). Differentially abundant taxa were defined as those that met a false discovery rate (FDR)-adjusted significance threshold ($q < 0.05$ for the urban cohort and $q < 0.001$ for the rural cohort).

- **Predictive Functional Profiling**

To predict the functional metabolic potential of microbial communities based on 16S rRNA data, functional profiling was performed using the q2-picrust2 plugin (v2023.9) within the QIIME2 framework. Utilizing the MetaCyc database, which contains information on metabolic pathways and enzymes. The resulting pathway abundance table (pathway_abundance.qza) was subjected to differential abundance testing using ANCOM-BC, implemented via the qiime2 composition

ancombc plugin. The official metaCyc_pathways_info.txt mapping file from the PICRUSt2 database was used to map predicted MetaCyc pathway identifiers to descriptive names. Differentially abundant pathways were defined as those that met a false discovery rate (FDR)-adjusted significance threshold ($q < 0.05$ for the urban cohort and $q < 0.001$ for the rural cohort).

References:

1- Callahan, B. J., McMurdie, P. J., Rosen, M. J., Han, A. W., Johnson, A. J. A., & Holmes, S. P. (2016). DADA2: High-resolution sample inference from Illumina amplicon data. *Nature Methods*, *13*(7), 581–583. https://doi.org/10.1038/nmeth.3869

2- Janssen, S., McDonald, D., Gonzalez, A., Navas-Molina, J. A., Jiang, L., Xu, Z. Z., Winker, K., Kado, D. M., Orwoll, E., Manary, M., Mirarab, S., & Knight, R. (2018). Phylogenetic Placement of Exact Amplicon Sequences Improves Associations with Clinical Information. *MSystems*, *3*(3). https://doi.org/10.1128/mSystems.00021-18

3- Vázquez-Baeza, Y., Pirrung, M., Gonzalez, A., & Knight, R. (2013). EMPeror: a tool for visualizing high-throughput microbial community data. *Gigascience*, *2*(1). https://doi.org/10.1186/2047-217X-2-16

4- Lin, H., Peddada, S.D. Analysis of compositions of microbiomes with bias correction. *Nat Commun* 11, 3514 (2020). https://doi.org/10.1038/s41467-020-17041-7
